# Experiences of patients and service providers with out-patient rehabilitation services in a rehabilitation centre in the Western Cape Province

**DOI:** 10.4102/ajod.v4i1.164

**Published:** 2015-12-10

**Authors:** Anne Kumurenzi, Charlyn Goliath, Gubela Mji, Nondwe Mlenzana, Conran Joseph, Sue Stathum, Anthea Rhoda

**Affiliations:** 1 Department of Physiotherapy, University of Rwanda, Rwanda; 2 Division of Physiotherapy, Stellenbosch University, South Africa; 3 Physiotherapy Department, University of the Western Cape, South Africa; 4 Karolinski Health Institute, Stockholm, Sweden

## Abstract

**Background:**

Rehabilitation is important for persons with disabilities as it contributes to their sense of autonomy, self-worth and social participation, and improves their quality of life. Improving the quality of rehabilitation services requires the dialogue of patients’ perceptions with those of service providers, in order to recommend informed reform.

**Objective:**

The objective was to explore the experiences of persons with physical disabilities and service providers, regarding the multi-disciplinary rehabilitation services provided at a community-based out-patient rehabilitation centre.

**Methods:**

A qualitative, exploratory study design was used to collect the data. A focus group was conducted with conveniently selected persons with physical disabilities. Three in-depth interviews were conducted with purposively selected key informants. All ethical considerations were adhered to during the implementation of the study.

**Results:**

Patients and service providers had different experiences regarding accessibility to rehabilitation services, and similar experiences with patient education and intensity of rehabilitation. Although the patients experienced that the service providers had sufficient knowledge and skills to manage them, services providers expressed that they lacked certain skills.

**Conclusions:**

The experiences expressed highlighted the need to improve rehabilitation services in terms of increasing the capacity of service providers and providing transport services for persons with disabilities.

## Background and literature review

The number of people living with a disability is increasing. The rate of disability ranges from 11.8% in higher income countries to 18.0% in lower income countries, in those aged 18 years and older (World Health Organisation 2011). Disability impacts negatively on the physical, psychological, mental, economic, and social well-being of individuals (Anderson *et al.* 2000; Mont 2007). In South Africa, the impact of disability is vast, which negatively affects persons with disabilities (PWDs), their families, communities and health services (Mont 2007). Rehabilitation is a vehicle that can be used to address the impact of disability as it is a process that assists with addressing the impact of disability on the lives of PWDs, by contributing to their sense of autonomy, self-worth and social participation (Eva & Wee 2010).

Furthermore, rehabilitation is a concept that is aimed at enabling PWDs, allowing them to reach and maintain their optimal physical, sensory, intellectual, psychological and social functional levels (United Nations 2007). Although rehabilitation is reported to improve the quality of life of PWDs, it has been estimated that amongst the 16% of people living with disabilities in Africa, only 2% have access to rehabilitation services (UN 2007). In contexts where rehabilitation services are lacking or inadequate, the life style of PWDs could be negatively affected.

Seeking to ascertain patients’ perceptions regarding rehabilitation programmes and services, is important for advocating change (DOH 2000). This is also important as clinicians and patients could have different views on outcomes, post-illness (Hewlett 2003). Patients’ experiences of a rehabilitation centre revealed that a relaxed environment, friendly and understanding staff and the presence of other patients contributes to a positive rehabilitation experience (Wain, Kneebone & Billings 2005). Individuals with disabilities have also reported that there is a lack of rehabilitation programmes that focus on facilitation of income-generating activities and access to schools or vocational training (Dusaberurema 2009). Service providers had also previously reported that they found it difficult to reach certain areas to provide community-based services to people with disabilities, resulting from geographical constraints (Dusaberurema 2009). Poor referral systems, a shortage of staff, long travelling distances and a lack of support were highlighted as the major challenges identified by mothers in the management of their children with a clubfoot (Kingau, Rhoda & Mlenzana 2015).

In South Africa, rehabilitation can be provided at in-patient settings, out-patient settings and, to a lesser extent, in the patients’ homes and communities. A few studies have reported on the PWDs’ experiences regarding rehabilitation services in the Western Cape, South Africa (De la Cornillere 2007; Kahonde, Mlenzana & Rhoda 2010). De la Cornillere (2007) reported on stroke patients’ experiences regarding stroke groups, whilst Kahonde *et al.* (2010) explored the experiences of persons with physical disabilities of rehabilitation services, at a number of community health centres. A consistent theme across these studies is that patients still encounter problems in accessing rehabilitation services in terms of transport, a shortage of resources and a lack of information provision regarding programmes and services of PWDs.

Although there were studies conducted at some community health centres in the Western Cape, the information is based only on patients’ experiences. According to Wottrich *et al.* (2004), exploring the perceptions of both service providers and patients proved to be crucial in improving physiotherapy services. Perceptions of service providers regarding experiences of rehabilitation are limited in the literature. Accessing persons with disabilities in the community has been highlighted as a factor-limiting rehabilitation identified by service providers (Dusaberurema 2009). In addition the local studies did not report on community-based rehabilitation services provided by a fulltime physiotherapist and occupational therapist with speech therapy services offered mainly by students. The rehabilitation services were, therefore, provided by a multidisciplinary team on a regular basis, as other community-based settings might have locally based fulltime physiotherapists, with part time or sessional occupational therapists and no speech therapist. Students can be seen as a vital component of the team, as rehabilitation services provided by students are rated as being high (Stiller, Sorich & Roberts 2013).

This paper provides the findings of a study that focused on the experiences of both the patients and the service providers to rehabilitation services, currently provided by a multi-disciplinary team at a rehabilitation centre in the Western Cape. The objectives of the study were to explore the perceptions and opinions of persons with physical disabilities, as well as services providers’ experiences regarding the following:

- service providers’ knowledge of the disability
- treatment and skills training
- interaction with service providers
- information obtained from the service providers
- accessibility to rehabilitation services
- patient participation and involvement in the rehabilitation
- the structure of rehabilitation sessions.

The objectives of the study are based on certain principles of community-based rehabilitation. An important goal of community-based rehabilitation is to empower people with disabilities, their families and communities. In doing so rehabilitation services need to be accessible and involve people with disabilities and their families in the rehabilitation process (WHO 2011).

## Research method and design

### Setting

The study was conducted at an outpatient rehabilitation centre in the Cape Town Metro health district. Rehabilitation services at this centre are provided by one full-time physiotherapist and one occupational therapist respectively. Part-time speech and language therapy services are also provided to patients with physical and psychosocial impairments and disabilities. The services rendered at this centre include preventive, promotive, curative and rehabilitation services. Health conditions that are treated at this centre include:

- orthopaedic
- neurological
- surgical
- gynaecology and obstetrics
- paediatric
- respiratory.

The occupational therapy department commonly provides services to between 59–154 patients, whereas physiotherapy services are rendered to between 65–325 patients per month. At the centre, speech therapy services are provided by students and between 20–120 patients utilise this service per month.

### Population and sampling

The study population consisted of patients with physical disabilities and service providers working at the out-patient rehabilitation centre. Patient records of those aged 18 years and above who received rehabilitation during 2009, were divided into groups and, thus, stratified according to the seven most common physical health conditions managed at the specific rehabilitation centre. The study was carried out during 2010, which means that the population of patients that received rehabilitation services during the preceding year (2009) was eligible for participation, as they had completed their respective programmes. This was also undertaken to limit the degree of recall bias, which is inherent to qualitative studies. The conditions were:

- spinal cord injuries
- head injuries
- strokes
- amputees
- fractures and dislocations
- osteoarthritis
- neuromuscular afflictions.

From the most common health conditions, three patients were conveniently selected to take part in the study. A total of 21 conveniently-selected patients were telephonically informed about the study and their consent was sought. In total, 11 consented to participate in a focus group discussion (FGD) which was later held at the rehabilitation centre. Of those, six were female and the mean of age of the participants was 54.82 years. Participants’ characteristics are presented in Table 1. Concerning the service providers, all three employees at the centre participated in one-on one in-depth interviews. All were female and their years of working experience ranged from seven months to 15 years.

**TABLE 1:** Patients’ characteristics.

Name	Age	Gender	Patient’s condition
P1	66	Female	Osteoarthritis
P2	47	Male	Rotator cuff repair
P3	54	Female	Rheumatoid arthritis
P4	55	Female	Frozen shoulder
P5	52	Female	Stroke
P6	77	Male	Osteoarthritis
P7	44	Male	Stroke
P8	52	Male	Frozen shoulder
P9	70	Female	Osteoarthritis
P10	26	Male	Amputation
P11	60	Female	Osteoarthritis

### Design

An exploratory descriptive qualitative methodology was used to collect the data. This design provides a comprehensive summarisation of experiences that deal with the *‘why’, ‘how’ and ‘what’* questions surrounding the experiences of rehabilitation of service users and providers. Further, this design is useful for hypothesis generation and theory development, as it leans toward the naturalistic approach of studying a particular phenomenon within contextual constraints (Neergaard *et al.* 2009; Sandelowski 2000).

### Data collection

In both the FGD and the in-depth interviews that lasted for approximately 45 minutes to an hour, the researcher used an interview guide to explore the experiences regarding rehabilitation services. The FGD with the patients explored the following:

- the service providers’ knowledge and skills training
- interaction between patients and service providers
- information obtained by patients
- accessibility to rehabilitation services
- patient participation and involvement in rehabilitation
- structure of rehabilitation services.

The focus group was only conducted once and lasted for approximately 45 minutes. The same semi-structured guide was used for the in-depth interviews with the service providers.

The service providers were interviewed at the rehabilitation centre at a time convenient for them. The interviews ranged from 45 minutes to one hour, the researcher conducted the interviews and took the field notes. Two trained research assistants assisted in conducting interviews in Afrikaans.

### Data analysis

The focus group discussion and the interviews were tape-recorded and transcribed verbatim. The data were thematically analysed, using content analysis as the strategy. The transcripts in Afrikaans were professionally translated from Afrikaans to English. The English transcripts were translated back into Afrikaans to verify accuracy of the information. The data were coded into pre-determined themes according to the objectives of the study and related literature. Initially, the transcripts were read a few times for familiarisation, to obtain a sense of the whole, and to become increasingly immersed in the data. During the first reading, open coding was applied to elicit all possible meanings of the texts. After open coding, sub-themes were created using patient-characteristic words (in-vivo) in order to stay close to the data and to fulfil the inductive approach of ensuring that the experiences of participants emerged from the data (Sandelowski & Barroso 2007). At this point, the lead author compared the main concepts, and identified sub-themes, with the findings of the contributing author (project leader), in order to cross-check the main findings.

### Trustworthiness

Four qualitative criteria for trustworthiness were applied in this study: credibility, transferability, dependability and confirmability (Lincoln & Guba 1985) for quality of the data (Shenton 2004). Member-checking for this study was undertaken to ensure credibility of the information obtained from the FGD and in-depth interviews. This was achieved by summarising the information from the field notes of the participants at the end of the in-depth interviews, and that of the FGD, to ensure the clarity of the information provided by the participants. A code-recode procedure of analysing the data were used to ensure the dependability of this study. Peer checks were conducted by the project leader, who had conducted qualitative studies herself and presented qualitative studies in seminars, at all stages of the analysis. The recorded interviews and discussions and their analyses were given to the project leader to ensure confirmability.

### Ethical considerations

Written informed consent was obtained from each participant prior to the FGD and in-depth interviews, and they were ensured of their right to withdraw from the study at any time. The information sheet and consent form were translated from English to Afrikaans for participants who were not fluent in speaking and reading English. In addition, the FGD participants verbally agreed that any information discussed in the focus group would be kept confidential. This made them conscious in advance of their ethical responsibilities. At the end of the FGD and in-depth interviews participants were again given the option to consent or to withdraw from the study.

To preserve the anonymity of the participants’ information, the researcher used pseudonyms to identify participants P1–P11, and service providers SP 1–3, throughout the study, thus, concealing the participants’ names and other personal information provided. All participants consented to have their interview recorded.

## Results

The themes were based on the participants’ experiences on the pre-determined thematic domains, such as:

- the service providers’ knowledge and skills training
- accessibility of services
- the interaction of patients with their service providers
- patients’ involvement and participation in rehabilitation
- provision of information
- structure and organisation of rehabilitation sessions.

### Theme 1: Perceptions of participants regarding service providers**’** knowledge and skills

Knowledge regarding disability and treatment, as well as skills training amongst service providers, were the primary issues that arose under this theme. The findings of the study revealed that some participants expressed the view that the service providers always knew what they were doing and the conditions they dealt with. They also expressed that at times service providers managed patients based on the diagnosis made by the doctor; ‘The people treating us here are well educated, they are very good’ (P2); ‘I have not been here for a while but I experienced they knew what was wrong with me’ (P6); ‘I always knew I would be fine, because I felt these people [*service providers*] knew what they were dealing with’ (P7).

Although most participants trusted the service providers’ knowledge, the service providers experienced that they were not knowledgeable enough to deal with all types of disabilities, as expressed by one of the service providers in the following quote: ‘I lack some knowledge on certain conditions, like paediatrics, stroke, that we normally see here at this centre’ (SP1); ‘We do not have the expertise in all domains, so sometimes we may refer the patients because of this’ (SP2).

In addition, some participants in the study experienced that their service providers were adequately skilled to deal with their conditions. Whilst others revealed that their service providers misdiagnosed their conditions, which influenced the treatment.

‘I came to be treated for my back. When I started doing the exercises I felt it was not my back, but my hip. Then I had to go back for x-rays’. (P9)

‘The same thing happened to me, they treated me for rheumatoid arthritis but it was not, it was osteoarthritis’. (P6)

### Theme 2: Participants**’** experiences with accessibility to and within rehabilitation centres

In both the FGD and in-depth interviews, there were some key issues that arose within this pre-determined theme. A lack of accessibility to suitable transport to attend the rehabilitation centre was expressed by both patients and service providers. Although the service providers thought that the rehabilitation was accessible resulting from the presence of ramps and rails patients still experienced having problems with accessibility at the centre. Participants in the current study expressed their concerns that were related to delayed and inaccessible transport to the rehabilitation facility, as expressed by participants:

‘The bus takes long to come. When it comes, we are already late for our appointments’. (P7)

‘… we don’t want to get wet during winter … if there was a car from the rehabilitation centre it would be a lot more comfortable and easier … then we would always make it to the appointment’ (P4)

‘… those benches in the waiting room are too low for patients to sit on’ (P9)

‘I don’t think patients struggle while accessing the centre because the gate is not far from the building. There is a ramp that wheelchair-users can use when coming in and out of the building. There is also a toilet handle for wheelchair-users while using the toilet’ (SP1)

### Theme 3: Participants**’** experiences with the interaction of service providers with patients

The participants’ experiences regarding their interaction with service providers were related to their being respected, appreciated and cared for. This was resulting from the fact that the service providers were able to provide time to relate to and communicate with them. The experiences of the participants in the current study were confirmed by their service providers’ responses when asked about their relationship with patients. This is illustrated in the quotations below.

‘… they are loving people and show respect towards the patients’ (P8)

‘They are always aware when you have pain and they react positively and loving throughout the treatment’ (P2)

‘I usually make sure that I am not only treating the patients throughout the session. I make time to relate to my patients so that I may avoid them seeing me as someone superior’ (SP1)

‘we make time for the patients, despite the workload. We try to see a patient as an individual and try to assist the patient the best we can. We talk to them regarding their disabilities’ (SP2)

### Theme 4: Participants**’** experiences with participation and involvement in rehabilitation

The participants and the service providers reported positive experiences with the patients’ participation and involvement in rehabilitation. The participants expressed that service providers gave them the opportunities to express personal goals for rehabilitation and also explained treatment procedures. The following quotations illustrate these experiences:

‘I had to sit with my therapist and tell her I want to be able to use my hand and build another room on to my house’ (P7)

‘… whenever I came here she (service provider) would ask me what I want to do’ (P10).

‘The first thing I do is that I ask my patients what they want to achieve before we start the treatment. Both the patient and I work to achieve what he/she wants’ (SPA).

‘I encourage patients to keep on exercising to keep them engaged in their rehabilitation, so that they can be able to do what they used to do before’ (SP3)

### Theme 5: Participants**’** experiences with provision of information

There was agreement between the services providers and participants’ experiences regarding the provision of information. Both reported that information was provided using visual aids. The participants also expressed experiencing information provided by the service providers as detailed and relevant to their condition.

‘The service provider talked and explained to me, using the x-ray, exactly what was the problem I had and where it was in the body’. (P1)

‘… before I started my treatment here, which is very good compared to other places (other facilities), the therapist explained to me what was wrong with me. I received a form which explained my problem’. (P10)

‘… a stroke patient, he/she will be provided with information regarding his/her condition and the exercises that he/she needs to do at home’. (SP2)

‘There are different posters up in the rehab centre where they can get more information. They ask for any other information they need. We usually help them. We also have health promotion talks. We invite different health promoters to come and teach our patients about different health risks like HIV/AIDS’. (SP)

### Theme 6: Participants**’** experiences with structure and organisation of rehabilitation sessions

The main aspects that were highlighted by both the patients and the service providers within this theme were length of sessions and appointment schedules. The participants wanted longer treatment sessions, whilst the service providers highlighted that they could not increase frequency and length of rehabilitation sessions resulting from a lack of staff.

‘I just want slightly longer sessions with them [*service providers*] (P4)

‘… and we need them [*service providers*] to add more days to see us because I have also heard other patients complaining about it’. (P7)

‘I sometimes need more time and capacity to book them (patients) in because we are under staffed’. (SP2)

‘… but during November and December … there are waiting lists for patients. There no students at the centre to help, but we try our best to accommodate all the patients even though it’s not easy’. (SP3)

## Discussion

The aim of the study was to explore the experiences of both patients and service providers with multi-disciplinary rehabilitation services at a community-based rehabilitation centre.

The current study findings revealed that some participants expressed that the service providers were knowledgeable in regard to the conditions they managed. These findings are consistent with those in a qualitative study conducted in Sweden by Wottrich *et al.* (2004) which reported that patients trusted their physiotherapists’ competencies.

Whilst service providers reported that they lacked knowledge and training in some disabilities, their patients demonstrated a level of trust in the competency and knowledge of their service providers. It is not unusual that service providers could develop some gaps in their scope of practice. A lack of knowledge by service providers about the patients’ disabilities could be the result of inadequate knowledge (Wottrich *et al.* 2004) and training (Darrah, Magil-Evans & Adkins 2002). Hence, training is one of the ways of developing service providers’ skills and competencies regarding patients’ conditions and treatment (Morrison, George & Mosqueda 2008). The appropriate, accountable and ethical response to this mandates that these service providers seek some remedial steps to address the gaps they have in their knowledge. The Health Professionals Council of South Africa requires health professionals to identify areas that require some improvement in their scope of practice, and to yearly renew their licences, which is linked to their attendance at refresher courses. It is important that service providers are knowledgeable and trained to manage patients, as this would affect the outcomes of the patients’ treatment (Al-Abdulwahab & Al-Gain 2003).

Both services providers and patients agreed that challenges existed with accessing the centre resulting from a lack of appropriate transport. The participants expressed that they had challenges related to limited availability and inaccessibility of transport. These findings are consistent with those of Kahonde *et al.* (2010) and De la Cornillere (2007), who reported that patients in the Western Cape, South Africa, encountered problems of inaccessible and inefficient transportation services to attend rehabilitation appointments. The lack of transport could lead to patients not attending appointments (UN 2007). The issue of transport points to a need for a coordinated response between the Departments of Health and Transport. A difference of opinion existed between the patients and service providers regarding accessibility within the facility. Service providers expressed the opinion that the centre was accessible but patients experienced transportation to the rehabilitation centre as a challenge. The presence of ramps and toilet handles could have resulted in service providers thinking that the centre was accessible. Rehabilitation service providers are required to make an audit of the accessibility of the environment. This audit should be conducted in consultation with persons with disabilities to ensure that their needs are met.

Both patients and service providers reported that a positive relationship existed between patients and service providers at the centre. The findings regarding the interaction of service providers with patients are consistent to those of Darrah *et al*. (2002) who reported on the perceptions of adults and adolescents and their families regarding service delivery. The findings of Darrah *et al.*’s (2002) study reported that patients were respected, cared for and supported across all service areas. Previous literature has reported that patients expressed experiencing service providers as unfriendly when they were not prepared to invest time to relate and have a conversation with them (Hills & Kitchen 2007).

Patients and service providers had positive opinions regarding the patients’ participation and involvement in rehabilitation. Patients in FGD reported that their service providers gave them opportunities to identify their goals and explained the procedures of the treatment. This was confirmed by the service providers in their interviews. This finding was in contradiction to findings in a study conducted by (Wottrich *et al.* 2004) who reported that physiotherapists claimed that patients were actively involved in the physiotherapy sessions, whilst the patients denied their involvement.

Both patients and service provides concurred that visual aids were used to provide patients with information about certain conditions. In addition participants also reported that they received most of the information they needed from their service providers. The information regarding the patients’ disability and the treatment provided was well explained by the service providers. The inclusion of education is an important component of patient management as it facilitates adherence to treatment and also decreases mortality and morbidity (Lynggaard *et al.* 2014). A lack of patient education could result in patients being misinformed and lacking knowledge about their disabilities (Leith, Phillips & Sample 2004).

Both patients and service providers expressed the need to increase time spent in rehabilitation. These findings are similar to those of Lopopolo (2001) and Wottrich *et al.* (2004), who reported that patients were concerned with the limited time they spent in therapy. In a study conducted in the UK, patients also encountered problems of long waiting periods. This affected both the patients’ health and their satisfaction with the rehabilitation services (Tod, Lacey & McNeill 2002). In South Africa primary healthcare services have been available to public health care users. Access to rehabilitation services, however, eludes the majority of disabled South Africans as it is characterised by congestion, long waiting times and staff who are overwhelmed by the congestion. It is in this regard that future rehabilitation research should focus on exploring the impact of family training and home based rehabilitation programmes, in alleviating patient load and improving patient care.

### Limitations of the study

This study presents some limitations. The study adopted focus group discussions which involved groups of people gathering the data related to the patients’ experiences regarding the rehabilitation services. The data were, therefore, not collected in a participant’s natural setting, which could have curtailed the freedom to speak freely. Furthermore, the targeted selection excluded those with speech and hearing impairments. For this reason the current study information cannot be generalised beyond the boundaries of the out-patient rehabilitation centres and the specific diagnostic groups. Future studies should include all representative diagnostic groups.

## Recommendations

Based on the design and limitations of the study, we identified the need for health-policy makers to improve the means of ensuring that rehabilitation facilities are adequately resourced to provide appropriate levels of quality care. On a macro-level, the results indicate that the government sectors in South Africa need to develop public transport systems that are accessible and affordable to persons with disabilities, to assist them to attend rehabilitation sessions. Increased home-based programmes could also be implemented to increase access to rehabilitation services.

## Conclusion

Patients and service providers had similar experiences regarding accessibility of rehabilitation services, patient education and intensity of rehabilitation. They differed with regards to knowledge and skills of service providers and accessibility within the rehabilitation centre. The experiences expressed highlighted the need to improve rehabilitation services in terms of increasing the capacity of service providers, and providing transport services for persons with disabilities.
